# The performance of computer-aided detection for chest radiography in tuberculosis screening: a population-based retrospective cohort study

**DOI:** 10.1080/22221751.2025.2470998

**Published:** 2025-04-22

**Authors:** Henan Xin, Wei Wang, Xiaomeng Wang, Juanjuan Huang, Yuanzhi Di, Jiang Du, Xuefang Cao, Boxuan Feng, Lingyu Shen, Yijun He, Tonglei Guo, Zihan Li, Jianguo Liang, Zhen Wang, Ping Zhu, Lei Gao

**Affiliations:** a NHC Key Laboratory of Systems Biology of Pathogens, National Institute of Pathogen Biology, and Center for Tuberculosis Research, Chinese Academy of Medical Sciences and Peking Union Medical College, Beijing, People’s Republic of China; b Key Laboratory of Pathogen Infection Prevention and Control (Ministry of Education), National Institute of Pathogen Biology, Chinese Academy of Medical Sciences & Peking Union Medical College, Beijing, People’s Republic of China; c Quzhou City Center for Diseases Control and Prevention, People’s Republic of China; d Zhejiang Provincial Center for Diseases Control and Prevention, People’s Republic of China

**Keywords:** Pulmonary tuberculosis, screening, computer-aided detection, chest radiography, early finding

## Abstract

From 2020 to 2022, a pulmonary tuberculosis (PTB) active case finding project based on chest X-ray (CXR) examination was conducted targeting individuals aged ≥65 years old in Jiangshan County, Quzhou City. The current study used computer-aided detection (CAD) software (JF CXR-1 v2) to retrospectively analyze the CXR images and to estimate its potential capacity for identifying PTB cases. The information of notified microbiologically confirmed PTB among the participants were exported from the Tuberculosis Information Management System. A total of 49,919 subjects participated in the 2020 examinations. Of these, 40,741 and 39,185 completed the follow-up surveys in 2021 and 2022, respectively. The pooled prevalence of suspected PTB reported by radiologists was 1.21% (1579/129,776), compared with 12.43% (16,129/129,776) reported by CAD. Of 101 bacteriologically confirmed PTB cases notified over three years, radiologists and CAD reported 45.54% (46/101) and 83.16% (84/101) as suspected cases, respectively. Among subjects with abnormal CAD (CAD score>0.35), the majority of the notified confirmed PTB patients (63/84) had their CAD scores >75% quantiles (as>0.75). With 3 years’ results, their CAD scores exhibited dynamic changes along with disease progression or treatment with median scores peaking in the year of diagnosis. This intriguing finding suggests that CAD for CXR reading assisted radiologists in PTB screening by reducing workload and improving case finding. The CAD primary score may have the potential to identify high-risk individuals and early PTB patients, adding a new dimension to our understanding of disease progression.

## Background

The targets of the End TB Strategy raised by the World Health Organization (WHO) are difficult to achieve, especially in high-burden countries, unless there are comprehensive strategies and intensive action work together. On average, one-third of the estimated 10 million individuals falling ill with tuberculosis annually remain undiagnosed or unreported to the WHO. Consequently, systematic screening for pulmonary tuberculosis (PTB) disease among high-risk populations is a crucial strategy aimed at ensuring early diagnosis [1]. Although chest X-ray radiography (CXR) has been used to screen or triage for PTB for several decades, in many undeveloped settings with a PTB epidemic, the lack of trained radiologist and the substantial intra- and inter-reader variability for CXR image interpretation is still big challenges faced [2,3]. With the development of artificial intelligence (AI), Computer-aided detection (CAD) for CXR image reading offers a potential tool to assist PTB imaging examination, especially in active case finding (ACF) applications. A study involving 23,954 individuals evaluated multiple AI algorithms as triage tests for PTB and showed that the five algorithms significantly outperformed experienced human readers in detecting tuberculosis-related abnormalities [4]. A recently published systematic review evaluated African studies using CAD as a screening tool to detect PTB against a microbiological reference standard. The results revealed a pooled sensitivity of 0.87 (95% confidence interval (CI), 0.78–0.96) and a specificity of 0.74 (95% CI, 0.55–0.93), very close to the WHO-recommended target product profile (TPP) for a screening test (sensitivity ≥0.90 and specificity ≥0.70) [5]. On this account, the WHO recommended CAD as an alternative to human interpretation of CXR for screening and triaging PTB in individuals aged 15 years or older in the latest consolidated guidelines [6]. Nevertheless, the recommendation was conditional due to the low certainty of evidence. The WHO calls for further evidence in regions with diverse population characteristics, disease presentation, different PTB prevalence, resource input, and economic level to more precisely evaluate the performance of CAD.

As a country with a high burden of PTB, China emphasized expanding the coverage of PTB ACF among key groups such as close contacts of PTB patients, elderly aged 65 years older, diabetes patients, HIV/AIDS patients, etc. CAD for CXR image reading has been gradually used to assist in PTB screening work in China since the first CAD software was approved in 2022. However, its application still lacks standardized guidance, and its performance needs systematic evaluation. In 2020, Jiangshan City in Zhejiang Province launched a 3 years’ PTB ACF project using CXR as a screening tool in rural elderly 65 years and older. Therefore, based on this real-world project, we conducted a study to retrospectively evaluate the performance of CAD for CXR reading in PTB screening and its potential value in early case finding.

## Methods

### PTB screening project conducted in Jiangshan City

The retrospective study was based on a three-year population-based PTB ACF project, using CXR as the initial screening tool, conducted in Jiangshan City, Zhejiang province. Jiangshan is located in the southeastern region of China, with an average notified PTB incidence of 69/100,000 from 2020 to 2022, about twice as high as the average rate of Zhejiang province. The PTB ACF project was supported by the Qu Zhou government and conducted between 2020 and 2020. The county-level hospitals and primary medical facilities in Jiangshan organized and implemented the screening examinations from March to October in each year. The project's target population comprises all persons (male and female) aged 65 years and above who reside in rural areas of Jiangshan City. For each participant, staff conducted a standard questionnaire and chest radiography examination. First, a standard questionnaire mainly containing information on the date of birth, gender, education, history of PTB history, and current suspected symptoms of active PTB was administered face-to-face by trained staff. Then, a digital posterior-anterior CXR was conducted. Two radiologists with experienced clinical practice from county-level hospitals, interpreted each participant's radiographs using standard criteria for reading results, adapted from the People's Republic of China Health Industry Standard (Classification of tuberculosis (WS196-2017)) [7]. The third superior radiologist would confirm any inconsistent CXR results. The final CXR results would be classified into one of the following four categories: (A) normal; (B) abnormal, suggestive of PTB; or (C) abnormal, suggestive of prior PTB; (D) abnormal, other lesions. Based on the above detection, any participants with radiographic abnormalities consistent with active PTB will be referred to a locally designated PTB hospital for further microbiological diagnosis. PTB diagnosis was made according to the National Health Industry Standard on Diagnosis for Pulmonary Tuberculosis (WS288-2017) [8].

### CAD reading the CXR retrospectively

The JF CXR-1 v2 software, developed by Jiangxi Zhongke Jiufeng Smart Medical Technology Co., Ltd., uses convolutional neural network algorithms for CAD readings in the current study. De-identified CXR images were uploaded uncompressed in DICOM format into cloud storage and analyzed by JF CXR-1 v2. The analysis process was automatic and blinded to other demographic and clinical data, including the final diagnosis. Results were finally provided as probabilistic scores, ranging from 0 to 1, which reflected the probability of active PTB or other lesions (increasing with abnormality). Apart from active PTB, JF CXR-1 v2 can simultaneously evaluate other thorax diseases, including prior PTB, pneumonia, tumours, pneumothorax, cardiac enlargement and pleural lesions based on different module. For each image, it has a score in each module. According to the manufacturer's instruction, 0.35 was the threshold for suspect PTB and 0.5 was the threshold for other lesions. CAD reading results were classified into one of four categories: for image with PTB module>0.35 were deemed as abnormal, suggestive of PTB; for image with PTB module≤0.35 and prior PTB module>0.5 were deemed as abnormal, suggestive of prior PTB; for image with PTB module≤0.35 and prior PTB module≤0.5 and tumours module≤0.5 and pneumothorax module≤0.5 and cardiac enlargement module≤0.5 and pleural lesions module≤0.5 were deemed as normal; for the rest individuals were deemed as abnormal, other lesions.

### Study design and population in current study

The target population of the current retrospective study was individuals who participated in the PTB ACF project and had available CAD results in Jiangshan County. Considering some subjects who participated in the project more than once, we included subjects who participated in the 2020 screening as the baseline study population and those who continue to participate in the 2021 or 2022 screening as the follow-up population. Using a retrospective cohort design, the study evaluated the performance of radiologists and CAD in identifying PTB cases and the value of CAD scores for early detection of high-risk PTB subgroups. The information of the microbiologically confirmed PTB cases registered in the Tuberculosis Information Management System (TBIMS) in Jiangshan from 2020-2022 was exported for the retrospective evaluation of the CAD's ability to identify PTB cases. PTB patients registered within 6 months after the screening were regarded as identified by the ACF project. The Institute of Pathogen Biology and the Chinese Academy of Medical Sciences Ethics Committees approved the current study (IPB-2024-12).

### Statistical analyses

The data were analyzed using SAS 9.4. The corresponding variables were treated as null values for participants with missing information during the analysis. Pearson's chi-square and Fisher's exact tests were used to compare the different distributions of the categorical variables. Chest results interpreted by radiologists and CAD were used alone or combined using “or,” “and,” or “step” to generate different algorithms. The ability to identify PTB cases using different algorithms was evaluated using bacteriologically confirmed PTB patients identified from TBIMS. For CXR identified as suspected PTB by JF CXR-1 v2 were regarded as subjects with abnormal CAD (CAD score>0.35). The primary CAD scores were divided into four classes according to quartiles. Tests for linear trend between score quartiles and the distribution or occurrence of PTB patients were conducted using Cochran–Armitage (chi-square) tests. The comparisons of median levels of scores for the same person in different years were conducted by Friedman tests and Dunn's multiple comparisons tests. The Kruskal–Wallis test compared median levels of scores for different groups. *p* < 0.05 was considered to reach statistical significance.

## Results

### Characteristics of the study subjects

**Figure 1.**
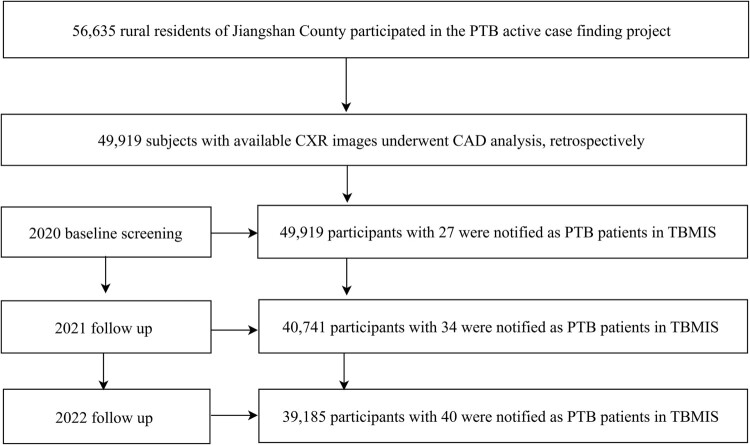
Flowchart outlining participant enrolment and follow-up process. CAD: Computer-aided detection; CXR: Chest X-ray; PTB: pulmonary tuberculosis; TBIMS: Tuberculosis information management system.

In 2020, a total of 56,635 individuals participated in the PTB ACF project in Jiangshan City. Of these, 49,919 subjects had qualified digital CXR images suitable for CAD analysis and were included in the current analysis. The follow-up surveys were completed by 40,741 participants in 2021 and 39,185 in 2022. In total, 101 subjects were identified as bacteriologically confirmed PTB cases in TBMIS (27 in 2020, 34 in 2021, and 40 in 2022) (Figure 1).

As shown in table 1, the median age of the 49,919 study subjects was 74 years. Approximately half of the participants (52.70%, or 23,613 out of 49,919) were male. A significant majority (84.12%, or 41,992 out of 49,919) had an education level below middle school, and nearly 60% (30,497 out of 49,919) had normal Body Mass Index (BMI). According to TBIMS, 137 participants had a record of prior PTB diagnosis within the past 5 years (2015–2019), but none experienced a relapse in current study.

**Table 1.** Characteristics of the study participants.

Variables	n* (%)
**Total**	49,919
**Sex**	
Male	23,613 (47.30)
Female	26,036 (52.70)
**Age (years)**	
65–<70	7509 (15.04)
70–<75	18,748 (37.56)
75–<80	12,601 (25.24)
≥80	11,061 (22.16)
Median (Q25-Q75)	74 (71–79)
**Education level**	
Illiteracy	14,681 (29.68)
Primary school	27,311 (55.22)
Middle school	7251 (14.66)
High school or higher	216 (0.44)
**BMI (kg/m^2^)**	
<18.5	5812 (11.64)
18.5–<24	30,497 (61.09)
24–<28	11,292 (22.62)
≥28	2318 (4.64)
**With history of microbiologically diagnosed PTB in past 5 years**	
No	49,782 (99.73)
Yes	137 (0.27)
**PTB notified in TBIMS from 2020 to 2022**	
No	49,818 (99.80)
Yes	101 (0.20)
Notified in 2020	27 (0.05)
Notified in 2021	34 (0.07)
Notified in 2022	40 (0.08)
**Subjects completed follow-up in 2021**	40,741 (81.61%)
**Subjects completed follow-up in 2022**	39,185 (78.50%)

BMI: body mass index; PTB: pulmonary tuberculosis; Q25 = 25% quantiles; Q75 = 75% quantiles; TBIMS: Tuberculosis information management system.

*Sum might not always be in total because of missing data.

### The performance of radiologist and CAD in identifying suspected PTB

**Figure 2.**
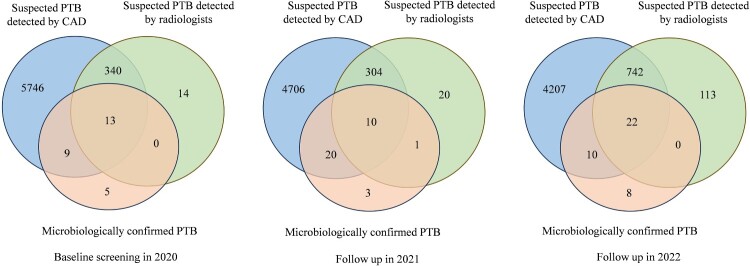
Venn diagrams showing the distribution of PTB cases identified by different algorithms. CAD identified suspected PTB cases are represented in blue, radiologist identified suspected PTB cases are represented in green, and microbiologically confirmed PTB cases are represented in orange. The numbers inside intersections represent overlap cases. CAD: Computer-aided detection; PTB: pulmonary tuberculosis.

As depicted in Figure 2, there are 0.74% (367/49,919), 0.82% (335/40,721), and 2.24% (877/39,136) participants were interpreted with suspected PTB by radiologists in 2020, 2021, and 2022, respectively. While the numbers of suspected PTB interpreted by CAD were much larger than that reported by radiologists, which is 12.24% (6,108/49,919) in 2020, 12.38% (5,040/40,721) in 2021, and 12.73% (4,981/39,136) in 2022, respectively. Among the suspected cases identified by radiologists, 3.54% (13/ 367) in 2020, 3.28% (11/335) in 2021, and 2.51% (22/877) in 2022 were diagnosed with microbiologically confirmed PTB. Their contribution to the notified PTB cases registered in the TBIMS was 48.15% (13/27) in 2020, 32.35% (11/34) in 2021, and 55.00% (22/40) in 2022, respectively. Among the suspected cases identified by CAD, 0.36% (22/6,108) in 2020, 0.60% (30/5,040) in 2021, and 0.64% (32/4,981) were diagnosed with microbiologically confirmed PTB. Their contribution to notified PTB was 81.48% (22/27) in 2020, 88.24% (30/34) in 2021, and 80.00% (32/40) in 2022, respectively (Table 2). During the 2021 follow-up, radiologists identified only one PTB patient as suspicious instead of CAD. As a result, no significant difference was observed when combining radiologists with CAD using “and” or “or” algorithms, compared to using either radiologist or CAD alone. Compared with completely relying on the interpretations of radiologists, the “step” algorithm “radiologist reading followed CAD” does not result in inferior performance, but saves at least 87% of work load for radiologists. For instance, in the 2020 screening, radiologists only need evaluate 6,108 CXRs with abnormal CAD rather than reviewing all 49,919 CXRs if CAD was adopted.

**Table 2.** The performance of radiologists and CAD on CXR based PTB screening among study population.

CXR based PTB screening algorithms	Numbers of CXR to be read n (%)	Numbers of reported suspected PTB n (%)	Percentage of confirmed PTB among the reported suspected cases % (n/N)	Contribution of CXR based ACF to PTB notification % (n/N)
**Baseline screening in 2020**				
Algorithm 1: Identified by radiologists	49,919 (100%)	367 (0.74%)	3.54% (13/367)	48.15% (13/27)
Algorithm 2: Identified by CAD	/	6,108 (12.24%)	0.36% (22/6,108)	81.48% (22/27)
Algorithm 3: Identified by radiologists or CAD	49,919 (100%)	6122 (12.26%)	0.36% (22/6,122)	81.48% (22/27)
Algorithm 4: Identified by radiologists and CAD simultaneously	49,919 (100%)	353 (0.71%)	3.68% (13/353)	48.15% (13/27)
Algorithm 5: Identified by radiologists among CAD abnormalities	6108 (12.24%)	≥ 353 (5.78%)	/	≥48.15% (13/27)
**Follow up in 2021**				
Algorithm 1: Identified by radiologists	40,721* (100%)	335 (0.82%)	3.28% (11/335)	32.35% (11/34)
Algorithm 2: Identified by CAD	/	5040 (12. 38%)	0.60% (30/5,040)	88.24% (30/34)
Algorithm 3: Identified by radiologists or CAD	40,721 (100%)	5061 (12.43%)	0.61% (31/5,061)	91.18% (31/34)
Algorithm 4: Identified by radiologists and CAD simultaneously	40,721 (100%)	314 (0.78%)	3.18% (10/314)	29.41% (10/34)
Algorithm 5: Identified by radiologists among CAD abnormalities	5040 (12. 38%)	≥314 (6.23%)	/	≥29.41% (10/34)
**Follow up in 2022**				
Algorithm 1: Identified by radiologists	39,136^#^ (100%)	877 (2.24%)	2.51% (22/877)	55.00% (22/40)
Algorithm 2: Identified by CAD	/	4981 (12.73%)	0.64% (32/4981)	80.00% (32/40)
Algorithm 3: Identified by radiologists or CAD	39,136 (100%)	5094 (13.02%)	0.63% (32/5094)	80.00% (32/40)
Algorithm 4: Identified by radiologists and CAD simultaneously	39,136 (100%)	764 (1.95%)	2.88% (22/764)	55.00% (22/40)
Algorithm 5: Identified by radiologists among CAD abnormalities	5094 (13.02%)	≥764 (14.99%)	/	≥55.00% (22/40)

ACF: active case fining; CAD: Computer-aided detection; CXR: chest X-ray radiography; PTB: pulmonary tuberculosis.

*PTB patients who had been registered in Tuberculosis Information Management System in 2020 were excluded; # PTB patients who had been registered in Tuberculosis Information Management System in 2020 and 2021 were excluded.

During the 3 years, 55 and 17 confirmed PTB patients were unreported as suspected cases by radiologists and CAD, respectively. They were more likely to be interpreted as “other pulmonary abnormality” by radiologists (54.55%, 30/55) and “prior PTB” by CAD (47.06%, 8/17) (Supplementary Table 1).

### The distribution of CAD scores among reported suspected PTB cases and confirmed PTB cases

Among subjects with abnormal CAD each year, each subject's CAD score was classified into four subgroups by quartiles. The distribution of CAD scores was found to be linearly correlated with the proportion of suspected PTB identified by radiologists and notified PTB cases (p for trend<0.001). Most suspected PTB patients reported by radiologist simultaneously had CAD scores exceeding the 75% quantiles (Q75) threshold. This trend was consistently observed in notified PTB patients, their CAD score predominantly focused on > Q75 subgroups, ranging from 70% (21/30) to 81·82% (18/22) (Table 3).

**Table 3.** Distribution of abnormal CAD scores among suspected PTB cases and notified PTB cases identified during follow-up.

CAD score	Suspected PTB cases reported by radiologists simultaneously n (%)	Notified PTB cases n (%)
**Baseline screening in 2020**		
**Total**	**353** (**100)**	**22** (**100)**
**Classified by quartiles**		
0.35 < score≤0.44	15 (4.25)	0 (0.00)
0.44 < score≤0.56	28 (7.93)	1 (4.55)
0.56 < score≤0.75	47 (13.31)	3 (13.64)
score>0.75	263 (74.50)	18 (81.82)
*p* for trend	<0.001	<0.001
**Follow up in 2021**		
**Total**	**314**	**30**
**Classified by quartiles**		
0.35 < score≤0.45	10 (3.18)	0 (0.00)
0.45 < score≤0.57	20 (6.37)	5 (16.67)
0.57 < score≤0.75	46 (14.65)	4 (13.33)
score > 0.75	238 (75.80)	21 (70.00)
*p* for trend	<0.001	<0.001
**Follow up in 2022**		
**Total**	**764**	**32**
**Classified by quartiles**		
0.35 < score≤0.45	55 (7.20)	1 (3.13)
0.45 < score≤0.57	72 (9.42)	2 (6.25)
0.57 < score≤0.76	158 (20.68)	5 (15.63)
score > 0.76	479 (62.70)	24 (75.00)
*p* for trend	<0.001	<0.001

CAD: Computer-aided detection; PTB: pulmonary tuberculosis.

Of 6086 subjects with abnormal CAD results that had not been diagnosed in baseline, 27 and 18 were identified as PTB patients in 2021 and 2022. There was a positive linear trend between baseline CAD scores and the incidence of PTB during follow-up (p for trend <0.001). Approximately two-thirds (64.44%, 29/45) of incident cases had baseline CAD scores higher than > Q75 as well (Table 4).

**Table 4.** The occurrence of PTB during follow up among subjects with abnormal baseline CAD.

Baseline CAD score in 2020	The occurrence of PTB in 2021 among subjects with abnormal baseline CAD	The occurrence of PTB in 2022 among subjects with abnormal baseline CAD
**Total**	27/6086*	18/6086*
**Classified by quartiles**	** **	** **
0.35 < score≤0.44	1/1494 (0.07)	2/1494 (0.13)
0.44 < score≤0.57	5/1486 (0.34)	0/1486 (0.00)
0.57 < score≤0.76	4/1606 (0.25)	4/1606 (0.25)
score > 0.76	17/1500 (1.13)	12/1500 (0.80)
*p* for trend	<0.001	<0.001

CAD: Computer-aided detection; PTB: pulmonary tuberculosis.

* PTB patients who had been registered in Tuberculosis Information Management System in 2020 were excluded.

### Changes in CAD scores over three years

**Figure 3.**
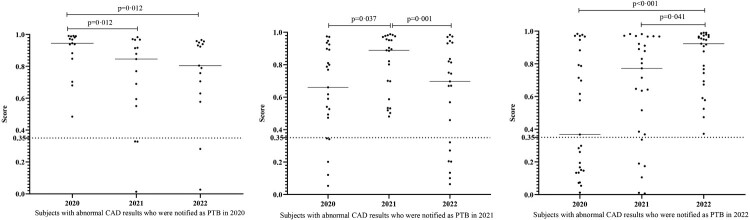
Changes in CAD scores among subjects with abnormal CAD who had been notified as PTB cases. The median CAD score was found to be statistically higher in the year of diagnosis and showed an increased or decreased trend depending on the progress or treatment of active disease. Horizontal lines represent median score levels. The comparisons of median levels of scores between different years were conducted by Friedman tests and Dunn's multiple comparisons tests. CAD: Computer-aided detection; PTB: pulmonary tuberculosis.

A total of 34,614 participants had CAD results over three years, and their demographic characteristics are displayed in Supplementary Table 2. Among those with abnormal CAD results, there were 15 PTB cases reported in 2020, 23 PTB cases in 2021, and 27 PTB cases in 2022. The changes in their CAD scores are illustrated in Figure 3. Notably, the median score was statistically higher in the year of diagnosis and displayed either an upward or downward trend depending on the progress and treatment of active disease.

## Discussion

Based on a large-scale population-based screening project, the current study retrospectively evaluated the performance of CAD for CXR reading in PTB early case findings in older people. Among 49,919 participants included in the analysis, 101 were notified with bacteriologically confirmed PTB in TBMIS over three-years. CAD was found to outperform radiologists by identifying 38 more PTB cases. Compared with “radiologist reading,” the step algorithms “radiologist reading followed CAD” might assist radiologists identifying more PTB cases and save the workload. Dynamic change of CAD scores along with disease progression or treatment with median scores peaking in the year of diagnosis was observed, which suggests the potential value in identifying early PTB cases or subgroups at high risk of developing PTB.

In view of its ease of implementation, rapid, simple, and high acceptability, CXR was still the dominant tool for PTB screening. However, the accuracy of CXR screening was influenced by radiologists’ ability to identify PTB cases. Usually, well-trained radiologists from primary-level health facilities are limited [9,10]. The advent of AI-based CAD provides a solution to the above challenges. In the current study, CAD identified 40% more PTB cases than human readers which indicated CAD might be capable of identifying subtle lesions consistent with early PTB, that were not visible to the human reader. Despite the improved sensitivity, most participants were overestimated as suspected cases. A recent meta-analysis synthesized the clinical evidence for the diagnostic accuracy of certified AI products designed for screening PTB in CXR compared to a microbiological reference standard and found the pooled positive predictive value (PPV) was only 0·19 (95%CI: 0.18-0.20) [11]. A preprint article also found that the evaluated three CAD systems (CAD4TBv6, qXRv2 and Lunitv4.9.0) had lower specificity than the expert radiologists [12]. Thus, using CAD alone as an initial screening tool would lead to a significant proportion of non-PTB patients under unwarranted further diagnostic tests. The trade-off between the number of cases identified and the proportion of microbiological tests that can be avoided must be considered in a real-word implementation. The step algorithms “radiologist reading followed CAD” might a solution to tackle the problem. Due to its high sensitivity, CAD was used as initial screening methods, and senior or expert radiologists will recheck those with abnormal CAD. Double positives will be referred for further microbiological tests. In mass PTB screening, the step algorithms could effectively decrease the workload of human readers while maintaining a high level of sensitivity and concurrently reducing false positive results [13]. Further analyses are needed to evaluate the cost-effectiveness of the entire process of different algorithms including both screening and subsequent diagnosis tests. In addition, given that most confirmed PTB patients are concentrated in the subgroups with the highest CAD scores, modifying the threshold of CAD based on the PTB epidemic of the target population and available of health resources is an approach to achieve the optimal performance of screening strategy in future applications. Other evidence also proved that optimized threshold scores vary for vulnerable populations and settings [14,15]. Still, the best to determine the thresholds in real-world CAD implementation needs further practice [16]. A pilot study to test participants’ sputum was suggested to capture the full spectrum of active PTB disease and provide unbiased data for determining an optimal threshold [17].

In agreement with the previous report [6,18,19] the CAD scores were significantly associated with the human readers’ results for CXR classification: the median of “suspect PTB,” “prior PTB,” “other pulmonary abnormalities,” and “normal” was 0.88, 0.24, 0.09, and 0.03, respectively. Furthermore, as far as we knew, dynamic changes in CAD scores corresponding with the progress or treatment of PTB were first observed in our retrospective cohort. In addition, most subsequent PTB incidence cases occurred among subgroups with the highest baseline CAD scores (>Q75). The results indicated a sustained increase in CAD score or a higher CAD score might indicate its potential value to predict PTB development. The exact mechanism was unknown, one explanation was that CAD exhibits an enhanced sensitivity in detecting incipient PTB manifestations compared to human reader, thus enabling earlier identification of disease onset. Another possibility was population who were accompanied with prior PTB lesions are inherently more susceptible to the reactivation or progression of the disease [20]. Our data support our hypothesis. On one hand, upon comparison, we found that among those with abnormal CAD outcomes, the average proportion of cases exhibiting prior PTB lesions as determined by human interpretation reached a noteworthy 42%, which is significantly higher than the proportion of 9% observed in the cohort without abnormal CAD results (Supplementary Table 3). On the other hand, the median level of CAD score was statistically higher among those with prior PTB history (median = 0.86) than those without (median = 0.56) (Supplementary Table 4). Thus, like previous research, which builds a radiograph scoring system by assigning numerical weights to specific features of chest radiographs consistent with PTB (such as cavitary lesions and upper lobe infiltrate) [21], the CAD score itself is a radiograph scoring system. A risk grading system for predicting PTB could be constructed by incorporating CAD scores and other parameters, such as demographics, clinical features, prior PTB history and close contact history [22]. Those ranked as high-risk should be attached to priority surveillance as the target population, annual or semiannual screening is necessary, which will facilitate the early diagnosis of PTB cases especially, those with sub-clinical features. For those ranked as low-risk, a rationalized screening schedule involving either a two-year or four-year interval appears practical [23].

There are several limitations in the study. First, the included study participants were only half of the target population in Jiangshan City. Considering that those more vulnerable to developing PTB might not be able to participate in the screening due to their poor health status, traffic inconvenience or unawareness of PTB, the performance of CAD might be influenced by selection bias. Strengthening the screening coverage rate is one important step for ensuring the effectiveness of screening in future studies. Second, the information of the confirmed PTB cases who had participated in ACF project came from TBIMS in current analysis, unreported cases due to referral failure or delayed diagnosis could not be completely avoided, which might affect the accuracy of the evaluation. Third, the current study only targeted the elderly 65 years old in Jiangshan City. CAD or radiologists’ PTB screening performance varied across regions and population characteristics. Therefore, it is necessary to carry out comprehensive evaluations in the different areas and populations to guide future CAD applications.

In conclusion, CAD for CXR assisted radiologists in identifying PTB cases in critical population screening applications. Furthermore, a sustained increase of CAD score in serial surveillance or a high level of CAD score (e.g. > Q75) along with other parameters might have the potential to predict early PTB, which provided us a new insight into identifying the target population who were under priority for PTB screening. Of course, current CAD products serving for CXR-based PTB screening require further performance improvement and algorithm optimization. Our findings require additional verification in a broader range of PTB screening scenarios.

## Supplementary Material

Supplementary material.docx

## Data Availability

The anonymised datasets used in this study can be available upon reasonable request to the corresponding author. Chest X-ray images will not be provided as these are withheld by the primary health facilities of Quzhou.
