# Supplementary material for: The performance of computer-aided detection for chest radiography in tuberculosis screening: a population-based retrospective cohort study
Source: Emerg Microbes Infect. 2025 Apr 22;14(1):2470998. doi: 10.1080/22221751.2025.2470998 (PMC12039420; doi:10.1080/22221751.2025.2470998)
Supplement: Supplementary material.docx [file TEMI_A_2470998_SM5417.docx]

**Supplementary material**

**Title: The performance of computer-aided detection for chest radiography in tuberculosis screening: a population-based retrospective cohort study**

**Short title:** The predictive value of CAD for CXR in PTB screening

Henan Xin PhD ^1,2^*, Wei Wang BMed^3^*, Xiaomeng Wang BMed^4^*, Juanjuan Huang MM*^1,2^, Yuanzhi Di BSc^1,2^, Jiang Du PhD ^1,2^, Xuefang Cao PhD ^1,2^, Boxuan Feng BSc ^1,2^, Lingyu Shen MM ^1,2^, Yijun He MM ^1,2^, Tonglei Guo MM ^1,2^, Zihan Li MSc ^1,2^, Jianguo Liang MM ^1,2,^ Zhen Wang MM ^4*^, Ping Zhu BMed ^3*^, Lei Gao PhD^1,2*^

1. NHC Key Laboratory of Systems Biology of Pathogens, National Institute of Pathogen Biology, and Center for Tuberculosis Research, Chinese Academy of Medical Sciences and Peking Union Medical College, Beijing 102629, P.R. China.
2. Key Laboratory of Pathogen Infection Prevention and Control (Ministry of Education), National Institute of Pathogen Biology, Chinese Academy of Medical Sciences & Peking Union Medical College, Beijing 102629, P.R. China.
3. Quzhou City Center for Diseases Control and Prevention, 324003, P.R. China.
4. Zhejiang Provincial Center for Diseases Control and Prevention, 310009, P.R. China.

* This authors contributed equally to this manuscript.

**Correspondence:** Mr. Zhen Wang, Zhejiang Provincial Center for Diseases Control and Prevention, 3399 Binsheng Road, Binjiang District, Hangzhou, 310009, China; Email: wangzhen@cdc.zj.cn; Mr. Ping Zhu, Center for Diseases Control and Prevention of Quzhou City, 154 Xi 'an Road, Quzhou City, 324003, China; Email: qzlyzp@126.com; Prof. Lei Gao, NHC Key Laboratory of Systems Biology of Pathogens, National Institute of Pathogen Biology, Chinese Academy of Medical Sciences and Peking Union Medical College, 16 Tianrong Street, Daxing district, Beijing, 102629, China. Email: gaolei@ipbcams.ac.cn.

**Supplementary Table 1 Chest results of confirmed PTB patients who are unreported as suspected PTB by radiologists or CAD**

| **Chest results** | **Unreported as suspected PTB by radiologist, N=55**  **n (%)** | **Unreported as suspected PTB by CAD, N=17**  **n (%)** | **p for fisher exact test** | |
| --- | --- | --- | --- | --- |
| Reported as normal | 8 (14.55%) | 5 (29.41%) | | 0.072 |
| Reported as prior PTB | 17 (30.90%) | 8 (47.06%) | |  |
| Reported as other pulmonary abnormalities | 30 (54.55%) | 4 (23.53%) | |  |

CAD=Computer-aided detection; PTB=pulmonary tuberculosis

**Supplementary Table 2 Demographic characteristics of study participants who participated in screening for three consecutive years**

| **Variables** | **n* (%)** |
| --- | --- |
| **Total** | 34,614 |
| **Sex** |  |
| Male | 15,788 (45.61) |
| Female | 18,826 (54.39) |
| **Age (years)** |  |
| 65-70 | 5,523 (15.96) |
| 70-75 | 13,717 (39.63) |
| 75-80 | 9,218 (26.63) |
| ≥80 | 6,156 (17.78) |
| **Education level** |  |
| Illiteracy | 9,752 (28.42) |
| Primary school | 19,443 (56.66) |
| Middle school | 4,990 (14.54) |
| High school or higher | 133 (0.39) |
| **BMI (kg/m^2^)** |  |
| <18.5 | 3,698 (10.68) |
| 18.5-24 | 21,182 (61.19) |
| 24-28 | 8,103 (23.41) |
| ≥28 | 1,631 (4.71) |
| **With history of microbiologically diagnosed PTB in past 5 years** | |
| No | 34,530 (99.76) |
| Yes | 84 (0.24) |
| **PTB identified by TBIMS during 2020 to 2022** | |
| No | 34,534 (99.77) |
| Yes | 80 (0.23) |

BMI= body mass index; TBIMS= Tuberculosis information management system

*****Sum might not always be in total because of missing data.

**Supplementary Table 3 Distribution of prior TB reported by radiologists classified by CAD results**

| **Variables** | **Individuals with abnormal CAD, %(n/N)** | **Individuals without abnormal CAD, %(n/N)** | **p forχ² test** |
| --- | --- | --- | --- |
|  | **Baseline screening in 2020** | |  |
| % of individuals reported as prior PTB by radiologists | 44.37% (2,710/6,108) | 9.36% (4,099/43,811) | <0.001 |
|  | **Follow up in 2021** | |  |
| % of individuals reported as prior PTB by radiologists | 46.19% (2,328/5,040) | 9.85% (3,513/35,681) | <0.001 |
|  | **Follow up in 2022** | |  |
| % of individuals reported as prior PTB by radiologists | 37.72% (1,879/4,981) | 8.64% (2,950/34,155) | <0.001 |

CAD=Computer-aided detection; PTB=pulmonary tuberculosis

**Supplementary Table 4** **Comparison of baseline median CAD score between people with and without prior TB history among those with abnormal CAD**

| **Variables** | **Individuals with prior TB history in past 5 years, N=107** | **Individuals without prior TB history** **in past 5 years, N=6,001** | **p for Kruskal-Wallis test** |
| --- | --- | --- | --- |
| The baseline median CAD score  (Q25-Q75) | 0.86  (0.62-0.92) | 0.56  (0.44-0.75) | <0.001 |

CAD=Computer-aided detection; PTB=pulmonary tuberculosis; Q25=25% quantiles; Q75=75% quantile
